# A highly conserved WDYPKCDRA epitope in the RNA directed RNA polymerase of human coronaviruses can be used as epitope-based universal vaccine design

**DOI:** 10.1186/1471-2105-15-161

**Published:** 2014-05-29

**Authors:** Refat Sharmin, Abul Bashar Mir Md Khademul Islam

**Affiliations:** 1 Department of Genetic Engineering and Biotechnology, University of Dhaka, Science Complex Building, Dhaka 1000, Bangladesh

**Keywords:** Coronavirus, SARS-CoV, MERS-CoV, RNA directed RNA polymerase, Epitope, Universal vaccine

## Abstract

**Background:**

Coronaviruses are the diverse group of RNA virus. From 1960, six strains of human coronaviruses have emerged that includes SARS-CoV and the recent infection by deadly MERS-CoV which is now going to cause another outbreak. Prevention of these viruses is urgent and a universal vaccine for all strain could be a promising solution in this circumstance. In this study we aimed to design an epitope based vaccine against all strain of human coronavirus.

**Results:**

Multiple sequence alignment (MSA) approach was employed among spike (S), membrane (M), enveloped (E) and nucleocapsid (N) protein and replicase polyprotein 1ab to identify which one is highly conserve in all coronaviruses strains. Next, we use various *in silico* tools to predict consensus immunogenic and conserved peptide. We found that conserved region is present only in the RNA directed RNA polymerase protein. In this protein we identified one epitope WDYPKCDRA is highly immunogenic and 100% conserved among all available human coronavirus strains.

**Conclusions:**

Here we suggest *in vivo* study of our identified novel peptide antigen in RNA directed RNA polymerase protein for universal vaccine – which may be the way to prevent all human coronavirus disease.

## Background

Coronaviruses are the diverse group of virus which infects domestic animals, birds as well as human [1]. Coronaviruses are enveloped viruses which are the members of Coronaviridae family [2]. Coronaviruses have positive strand RNA genome which is approximately 26–32 kb long. The overall structures of all coronaviruses are composed of the spike (S), envelope (E), membrane (M) and nucleocapsid (N) protein. The other non-structural proteins like RNA directed RNA polymerase, helicase, 3CL like proteinases etc are produced by the cleavage of replicase polyprotein 1ab or ORF 1ab polyprotein [3].

The first coronavirus 229E was identified in 1960. Since then different types of coronaviruses have emerged. HCoV-229E, HCoV-OC43, SARS-CoV, HCoV-NL63, HCoV-HKU1 and MERS-CoV are the coronaviruses which infect human [4]. Most of the coronaviruses cause respiratory, enteric, hepatic, or neurological diseases with highly variable severity in their hosts [5]. The first two coronavirus HCoV-229E and HCoV-OC43 infects lower respiratory tract where the later SARS-CoV, HCoV-NL63, HCoV-HKU1 infect both lower and upper respiratory tract [4,6-8]. But the newly emerged MERS-CoV infects both lung and kidney that reflects how these viruses are changing their cell tropism and becoming highly pathogenic [9].

Most infections caused by human coronaviruses were relatively mild. Among all the human coronaviruses, SARS-CoV and MERS-CoV are much more deadly. The severe acute respiratory syndrome (SARS) outbreak caused by SARS-CoV in 2002 to 2003. The SARS-CoV created outbreak resulted in a total of 916 deaths among more than 8000 confirmed cases in over 30 countries [10,11]. The newly emerged MERS-CoV is now posing a great threat for human. According to WHO, 75 people have died among more than 178 confirmed cases caused by MERS-CoV [4]. Though this MERS-CoV virus was first found in Saudi Arabia 2012, now it has been emerged in UK, France, Tunisia, Spain and Italy that indicates it’s going to create another outbreak like SARS-CoV [12-14]. From 1960 to till now there is no recommended drug or vaccine for MERS-CoV infection and treatment relies on exclusively supportive care, which gives the high case-fatality rate, is not highly effective [15].

In 2003 after the discovery of SARS-CoV, there were a significant increase in research on coronavirus, but no definitive antiviral or therapeutic treatment for coronavirus infections came from these researches [16]. From the clinical experience of SARS-CoV found that a number of interventions including ribavirin with and without corticosteroids, ribavirin with protease inhibitors and interferon with corticosteroids may improve outcome. But a definitive treatment was not clearly established and the therapeutic interventions have not been evaluated *in vivo*[17].

The identification of therapeutics is a high priority and though there is currently no specific therapy or vaccine for human coronaviruses, this disease has been severe with a high case-fatality rate [18]. As these viruses are now becoming pathogenic and causing outbreaks, so steps have to be taken to prevent human death. Vaccination is one of the most efficient ways to prevent infectious disease [19]. Effective vaccines controlling virus spread and disease are available for a number of infections, such as smallpox, poliomyelitis, measles, mumps, rubella, influenza, hepatitis A, and hepatitis B [20]. For coronavirus, this vaccine approach is hindered by the fact that human coronavirus strains are not structurally related and they are changing rapidly by recombination [21]. Therefore, designing a universal vaccine against conserved regions for all human coronaviruses is a major challenge at present.

With the disclosure of huge sequence information, epitope based vaccine design now has become a most promising approach for viral vaccine preparation [22]. In order to prepare vaccines, computational prediction of epitopes and vaccine design can reliably aid this process to reduce time and cost. Although the epitope based vaccine design is now a familiar concept, not much work has been done in case of coronaviruses.

In this study, we design an epitope based universal vaccine which can be use to prevent all kind of human coronaviruses. For this, bioinformatics analyses of viral proteins were done for finding the conserved peptide region and for mapping the evolutionary conserved epitope. The 3D structure of RNA directed RNA polymerase was determined by threading modeling technique and a highly immunogenic, accessible and conserved epitope was identified. This epitope can be used as a universal vaccine against all human coronaviruses.

## Results

### RNA directed RNA polymerase is highly conserved in all human coronavirus strains

**Figure 1:**
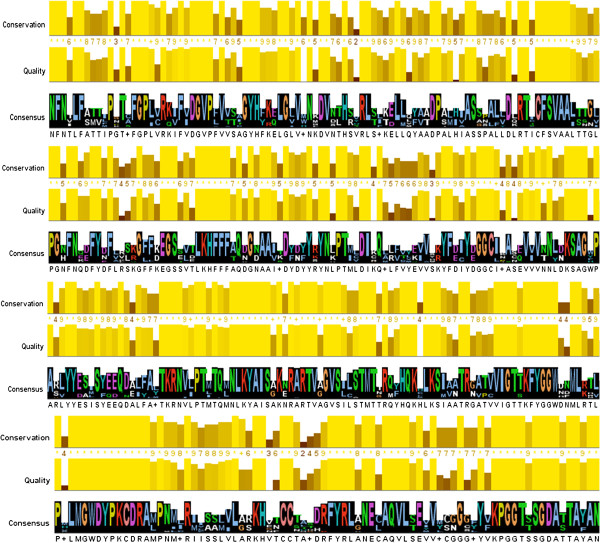
**Conserved peptide in RNA directed RNA polymerase.** Multiple sequence alignment of the 46 replicase polyprotein 1ab of all coronaviruses revealed that human coronaviruses are conserved in RNA directed RNA polymerase. This alignment was visualized by Jalview 2.8 [25]. Along the alignment this tool provides a graphical (bar chart) conservation summery using 11-base scale for conservancy and BLOSUM 62 for quality. For Conservation yellow color bar and star sign indicates the full conservation. Black bars showed the consensus sequence and yellow color indicates good quality. All the colors changes according to the conservation and alignment quality.

**Figure 2:**
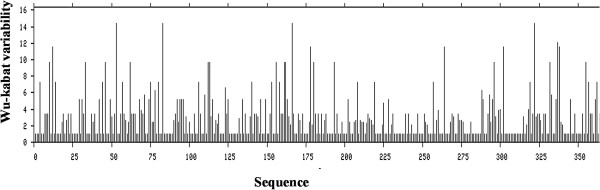
**Protein variability index of the conserved peptide.** The protein variability index of the conserved peptide was determined by using PVS server [24]. The conservancy threshold was 1.0 in this analysis. X axis indicates the amino acid position in sequences and Y axis indicates the Wu-kabat variability.

To find a conserved region, MSA by clustalW [23] and protein variability index [24] analyses were performed. No conserved region was found in case of S, E, M and N proteins (Additional file 1: Figure S1, Additional file 2: Figure S2, Additional file 3: Figure S3, Additional file 4: Figure S4 respectively). From the MSA of replicase polyprotein 1ab coronaviruses were found to be conserved in RNA directed RNA polymerase (Additional file 5: Figure S5). MSA of this RNA directed RNA polymerase region (Figure 1) and protein variability index (Figure 2) identified a 385 amino acid long conserved region among all human coronaviruses. The conserve sequence was then used to determine immunogenicity.

### Both YPKCDRA and YYVKPG identified as consensus and highly immunogenic epitopes by two different algorithms

**Figure 3:**
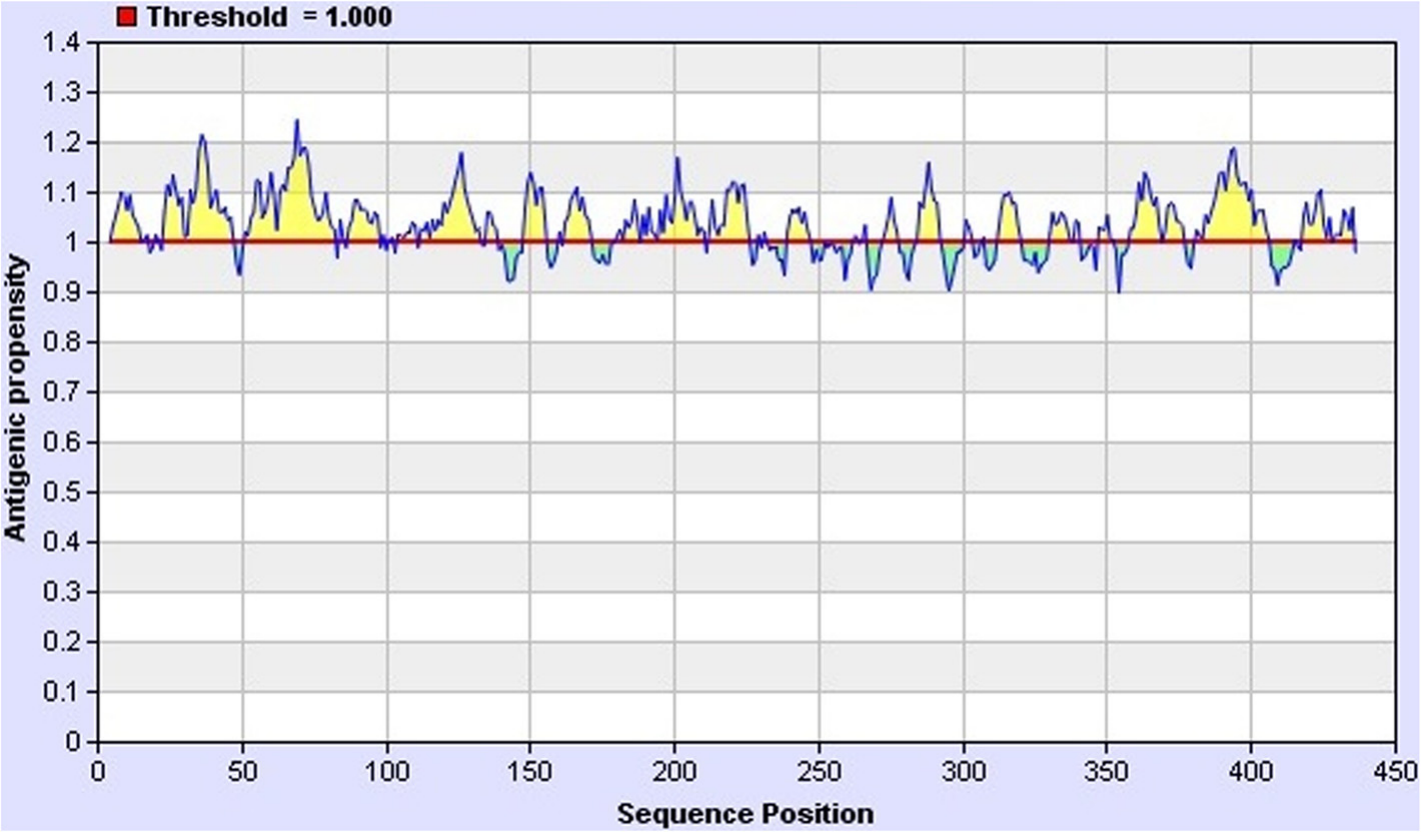
**Antigenicity of the conserved peptide.** The conserved peptide was found to be highly antigenic in the IEDB analysis [27]. Most of the residues were found above the threshold 1.00. Residues in the yellow region are antigenic and in the green region are below the threshold (red line).

For vaccine design the peptide has to be immunogenic and antigenic [26]. The conserved peptide was found to be highly antigenic (Figure 3) in IEDB epitope prediction analysis [27]. In this analysis 1.000 threshold was used and most of the residues in the peptides were found above the threshold level. B-cell epitopes were predicted using Immune Epitope Database (IEDB) [28] B-cell epitope prediction tool and Bepipred [29] using the conserved protein sequence. Several epitopes were predicted (Table 1) by these algorithms, but only those epitopes sequences that are found full or at least 90% overlap between IEDB B-cell epitope prediction tool [28] and Bepipred prediction [29] are chosen as desired epitopes (Table 2). YPKCDRA and YYVKPG epitopes were found to be consensus among both tools predicted epitopes.

**Table 1:** **Predicted antigenic sites and their lengths using Bepipred **[29]**and IEDB **[28]**analysis tools**

**Bepipred analysis**		**IEDB analysis**	
**Peptide**	**Length (aa)**	**Peptide**	**Length (aa)**
VRPGNFNQ	8	DYKVQLFEKYFKY	13
GNAAIT	6	HANCVNCTDDRCVLHCANFNVLFAM	25
DKSAGHPFN	9	KTCFGPIVRKIFVDGVPFVVSCGYHYKELGLV	32
SYQEQ	5	VSLHRHRLSLK	11
DVDNP	5	ADPAMHI	7
YPKCDRA	7	SSNAFLDLRTSCFSVAALTTG	21
YYVKPGGTSSGDATTAY	17	YDFVVSKG	8
		SVTLKHFFFA	10
		DYNYYSYN	8
		PTMCDIKQMLFCMEVVNKYFEI	22
		DGGCLNASEVVVNN	14
		KARVYYESM	9
		LKYAIS	6
		TVAGVSILS	9
		GATCVIGTT	9
		LKTLYKDV	8
		YPKCDRA	7
		CRIFASLILARKHGTCCTT	19
		YRLANECAQVLSEYVLCGGGYYVKPG	26
		ANSVFNILQA	10
		TANVSAL	7

**Table 2:** **Consensus antigenic sites between Bepipred **[29]**and IEDB analysis **[28]**predicted antigenic sites**

**Consensus Epitope**	**Length (aa)**
YPKCDRA	7
YYVKPG	6

### Nine surface accessible epitopes were predicted from the conserved peptide

**Figure 4:**
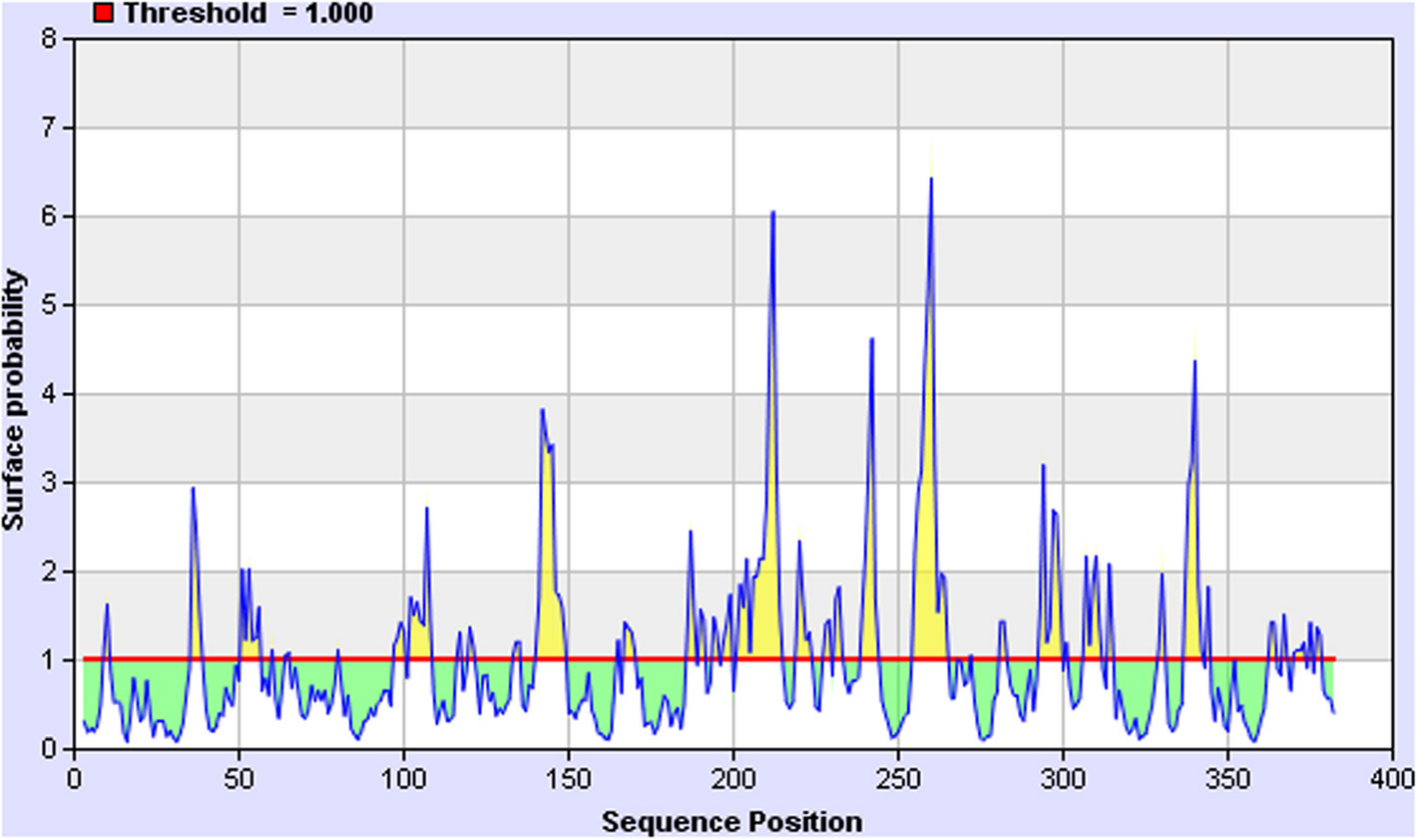
**Conserved peptide’s surface accessibility.** The surface accessible residues of the conserved peptide which are above the cut off are located in the yellow region. The red horizontal line indicates surface accessibility cutoff (1.000).

To become a vaccine, an epitope should be accessible to the antibody. If the antibody can bind to the epitope that will be able to induce an immune response [26]. The surface accessibility of the conserved peptide (Figure 4) was determined using 1.000 threshold level and nine accessible epitopes were found to be above the threshold level (Figure 4) (Table 3) using Immune Epitope Database (IEDB) [28] Emini surface accessibility prediction analysis [30]. Among these nine epitopes, WDYPKC epitope overlaps with the Bepipred [29] and IEDB [28] predicted consensus epitope YPKCDRA.

**Table 3:** **Predicted surface accessible antigenic sites by using Emini surface accessibility prediction analysis **[30]

**Peptide**	**Length (aa)**
HRHRLS	6
GNFNQDF	7
TDYNYYSYNL	10
ARVYYESMSYQEQD	14
AKNRAR	6
MTNRQYHQKML	11
TLYKDVD	7
WDYPKC	6
TRDRFY	6

### WDYPKCDRA is fully conserved among all human corona virus isolates

**Figure 5:**
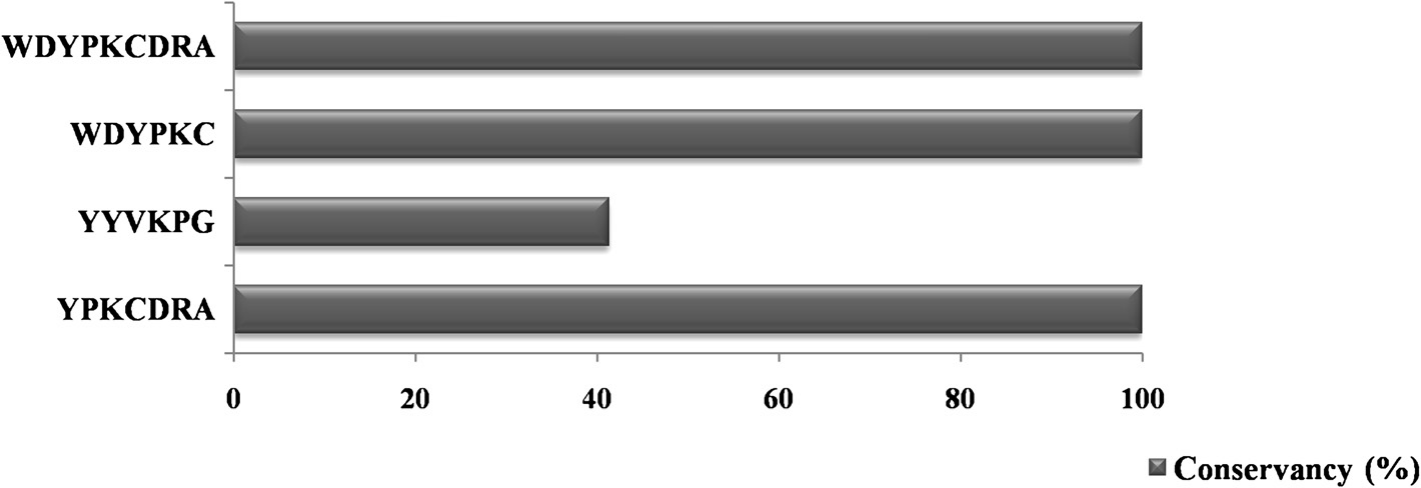
**Conservancy of the predicted consensus epitopes.** Three of the four epitopes were found to be 100% conserved. Here Y axis indicates the epitopes and X axis indicates the conservancy percentage.

The conservancies of all epitopes were determined by IEDB conservancy analysis tools [31]. From the IEDB predicted epitopes, two epitopes (YPKCDRA, LKYAIS) and from Bepipred predicted epitopes, YPKCDRA epitope were found to be 100% conserved among all human coronavirus isolates (Table 4). WDYPKC epitope from the surface accessible epitope was also found to be 100% conserved (Table 4). Among the two consensus epitopes of Bepipred [29] and IEDB [28] analysis, YPKCDRA epitope was found to be 100% conserved among all human coronavirus isolates (Figure 5). This YPKCDRA and WDYPKC epitopes are in the same region and 100% conserved in all human coronaviruses. Therefore, the whole epitope WDYPKCDRA which is 100% conserved was then selected as the desired universal vaccine candidate.

**Table 4:** **Predicted conservancy of the antigenic site by using IEDB conservancy analysis **[31]

**Sequence**	**Identity %**
**Bepipred analysis**	
VRPGNFNQ	17.39
GNAAIT	41.30
DKSAGHPFN	17.39
SYQEQ	17.39
DVDNP	41.30
YPKCDRA	100.00
YYVKPGGTSSGDATTAY	17.39
**IEDB analysis**	
DYKVQLFEKYFKY	17.39
HANCVNCTDDRCVLHCANFNVLFAM	17.39
KTCFGPIVRKIFVDGVPFVVSCGYHYKELGLV	17.39
VSLHRHRLSLK	17.39
ADPAMHI	17.39
SSNAFLDLRTSCFSVAALTTG	17.39
YDFVVSKG	17.39
SVTLKHFFFA	17.39
DYNYYSYN	17.39
PTMCDIKQMLFCMEVVNKYFEI	17.39
DGGCLNASEVVVNN	17.39
KARVYYESM	17.39
LKYAIS	100.00
TVAGVSILS	41.30
GATCVIGTT	17.39
LKTLYKDV	17.39
YPKCDRA	100.00
CRIFASLILARKHGTCCTT	17.39
YRLANECAQVLSEYVLCGGGYYVKPG	17.39
ANSVFNILQA	17.39
TANVSAL	17.39
**Surface accessibility prediction analysis**	
HRHRLS	17.39
GNFNQDF	41.30
TDYNYYSYNL	17.39
ARVYYESMSYQEQD	17.39
AKNRAR	56.52
MTNRQYHQKML	17.39
TLYKDVD	17.39
WDYPKC	100.00
TRDRFY	17.39

### WDYPKCDRA is also an accessible epitope

**Figure 6:**
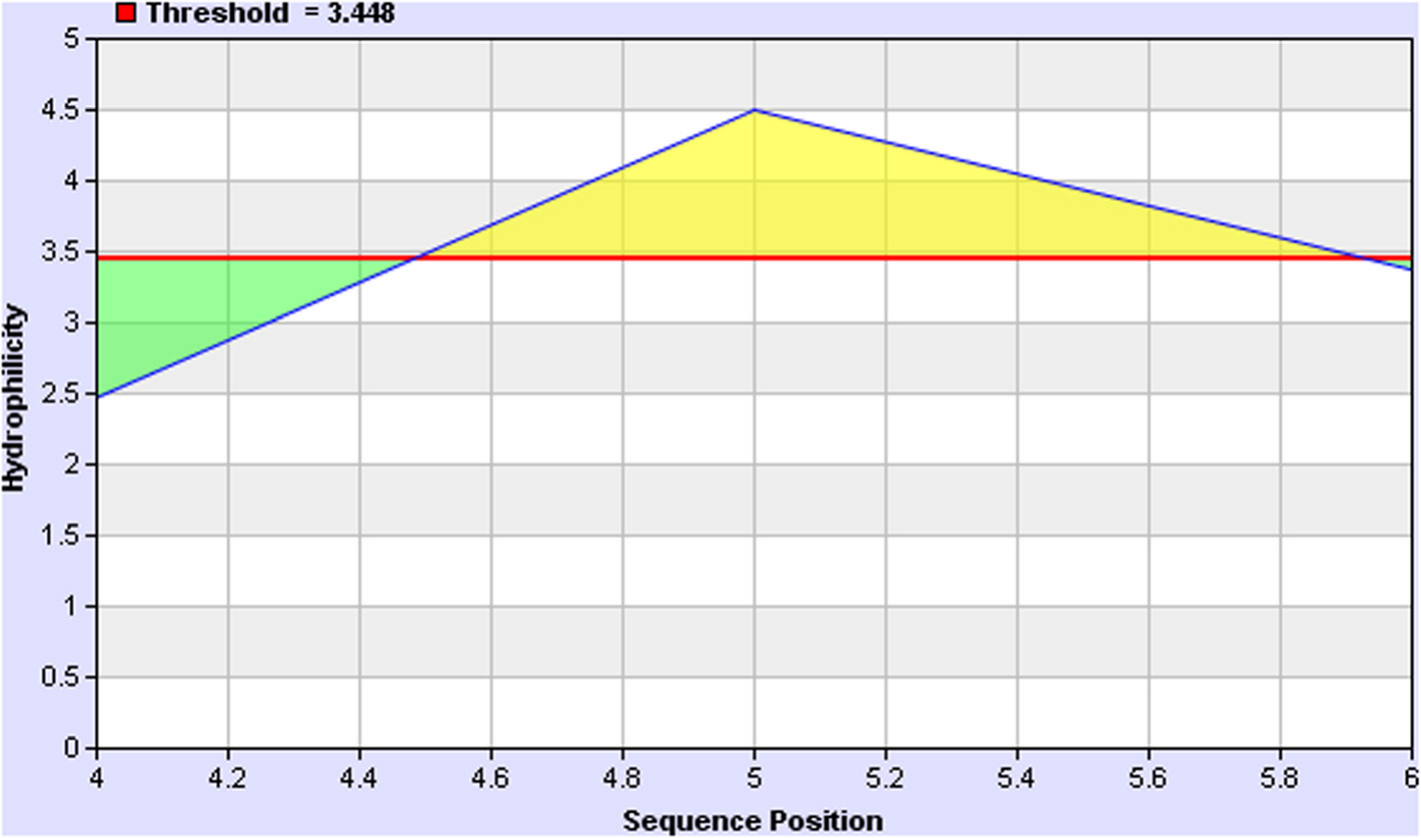
**Hydrophilicity of the WDYPKCDRA epitope.** Most of the residues of the desired WDYPKCDRA epitope were found to be hydrophilic in nature (in the yellow colored region). The residues which are below the cut off 3.448 (red line) are in the green region.

Hydrophilicity is desired feature of B cell epitope which indicates the accessibility of the epitope. The WDYPKCDRA epitope was found to be hydrophilic (Figure 6) in nature as determined by the IEDB Parker hydrophilicity analysis [32]. A threshold of 3.448 was used which is indicated by the red line and the residues of the epitope which are hydrophilic are in the yellow region. The maximum level was found as 4.5 (Figure 6) in the epitope.

### A tertiary structure of RNA directed RNA polymerase was predicted and validated using *in silico* approach

**Figure 7:**
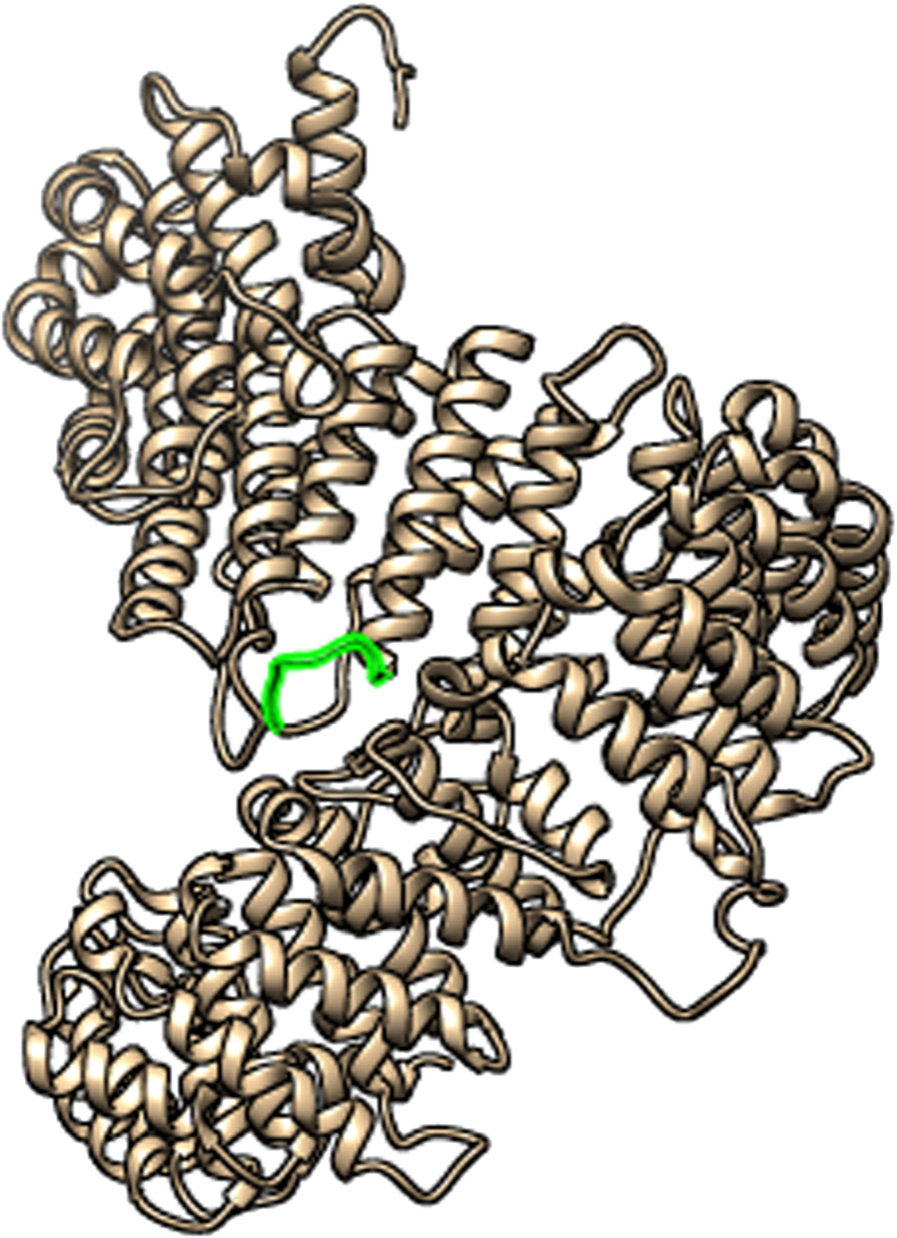
**3D structure of RNA directed RNA polymerase protein.** Predicted conserved WDYPKCDRA epitope mapped onto protein 3D structure using UCSF Chimera [35] visualization tool. Here green colored region indicates the conserved epitope WDYPKCDRA.

As the experimental tertiary structure of the RNA directed RNA polymerase is not available, we modeled a 3D structure by I-TASSER server [33] by multiple threading alignments. I-TASSER analysis deduced 5 different models (data not shown) for this protein. The quality of all the predicted protein models was checked by PROCHECK analysis [34]. From the PROCHECK analysis results, the protein model in which maximum numbers of amino acids residues were in maximum favorable region and G factor was highest was taken as the desired best model. The model in which 89.3% residues are found to be in the most favored region in Ramachandran plot (Additional file 6: Figure S6) and G-factor was -0.31 from the PROCHECK analysis was selected as the desired model. Along with the surface accessibility analysis and hydrophilicity analysis, the targeted WDYPKCDRA epitope was also found to be in the surface and accessible in the RNA directed RNA polymerase 3D structure (marked as green color) (Figure 7).

## Discussion

Coronaviruses are one of the most diverse groups of virus which are becoming a deadly virus day by day. Though the first two strains were not so much deadly but the other members were pathogenic. After SARS outbreak, a new coronavirus strain called MERS-CoV is now going to cause another outbreak [4]. The cell tropism and cellular receptor of the six types of coronaviruses are not similar (Additional file 7: Table S1). Though at first it was thought that SARS-CoV and MERS-CoV are structurally similar and tried to treat MERS-CoV infected patient with the SASR-CoV treatment. But it was found that they bind to two different receptors, namely ACE2 and DPP4 or CD26 respectively [36]. These viruses are actually zoonotic origin, undergo recombination, and may be in future another strain of this group of virus will come [37]. Therefore, it is important to take preventing measures not only to prevent this new strain of coronavirus, also against all the strain of coronavirus. There is no recommended vaccine for coronaviruses which is necessary to prevent. Most of the cases, vaccines were designed by targeting spike protein. Similarly, researchers also reported to design vaccine against SARS-CoV and MERS-CoV spike protein [38,39]. Fernando et al. also designed lived-attenuated MERS coronavirus by mutating MERS-CoV envelope protein as a vaccine which will be for only MERS-CoV [40]. These vaccines would thus be effective for only those strains not for others. Giving a universal vaccine for all strain of viruses is much more promising solution rather than giving individual vaccine for individual strain.

The concept of prevention of viruses by designing a universal vaccine has also been reported previously, for example against Influenza virus. In case of influenza virus, universal vaccine against matrix 2 protein which was found to be conserved among all Influenza subtypes was reported [41]. An attempt to design universal vaccine against members of coronaviruses, like feline infectious peritonitis (FIPV), canine coronavirus (CCV), gastroenteritis coronavirus (TGEV), bovine coronavirus (BCV) targeting their spike protein in 1993 were observed [42]. But this concept was not applied to human coronaviruses.

Vaccine development has been one of the most important contributions of immunology to public health to date. Traditionally vaccines were based on the intact pathogen, either inactivated or live attenuated. These types of vaccines had some crucial drawbacks like safety consideration and the loss of efficacy due to the genetic variation of the pathogen. But now a day’s these vaccine concepts are greatly replaced by novel vaccine approaches like naked DNA vaccine, epitope based vaccine. The main benefit of immunization with an epitope-based vaccine is the ability to immunize with a minimal structure and it will stimulate an effective specific immune response, while avoiding potential undesirable effects [43].

In this study, we aimed to design an epitope-based universal vaccine for all human coronavirus strain. For this purpose we did multiple sequence alignment of the spike (S), envelope (E), membrane (M), nucleocapsid (N) protein and replicase polyprotein 1ab of all six human coronaviruses. Replicase polyprotein 1ab was taken to check whether there is any conservancy among the non structural protein as this replicase polyprotein cleaved into 15 non-structural proteins. In case of S, E, M and N protein, no putative conserved region was found. But conserved region was found in case of replicae polyprotein 1ab where the conserved region was in the RNA directed RNA polymerase. This indicates that this protein is less mutating than the S, E, M and N protein. This RNA directed RNA polymerase protein was targeted to determine antigenic sites based on immunogenicity and surface accessibility using different bioinformatics analyses. The consensus antigenic sites were the desired one and their conservancy was also determined. From the conservancy analysis it was found that Bepipred [29] and IEDB [28] analysis predicted consensus epitope YPKCDRA and the surface accessibility analysis [30] predicted epitope WDYPKC are 100% conserved. This 100% conserved WDYPKC and YPKCDRA are actually located in the same region of RNA directed RNA polymerase and it was then taken as the targeted epitope. This epitope was found to be accessible and hydrophilic which is one of the crucial requirements for an epitope to be used as a vaccine. This reflects a promising scope to use this conserved epitope as a universal vaccine both as preventive and therapeutic treatment. As this epitope remain long been conserved since 1960, it may be possible to use this vaccine in future for upcoming human coronavirus strains as well. To become an effective vaccine, it needs to be highly immunogenic, stable inside the body. If it is poorly immunogenic or unstable, it needs to be conjugated with adjuvant [44]. Though this epitope based vaccine is designed by *in silico* analyses, the actual immunogenicity, stability, efficacy and their delivery strategy inside the recipients body can’t be determined by this *in silico* analysis. To address these questions *in vitro* and *in vivo* experiments are essential.

## Conclusions

This study shows that though the human coronaviruses are not structurally related but it is possible to design an epitope-based universal vaccine for all human coronavirus strains. Our results are based on sequence analysis and computational predictions show predicted epitope would be a candidate target for the universal vaccine; and to determine the actual effectiveness of the peptide for mounting an immune response both *in vitro* and *in vivo* studies can be performed.

## Methods

### Retrieving coronavirus structural and nonstructural protein sequences

A total available 46 replicase polyprotein 1ab, 17 spike (S) protein, 18 envelope (E) protein, 18 membrane (M) and 18 nucleocapsid (N) protein sequence data were retrieved from NCBI GenBank sequence database [45] (Additional file 7: Table S1).

### Identification of conserved region

To find the conserved region, retrieved sequences were aligned using EBI-clustalW program [23]. This multiple sequence alignment (MSA) was done with Gonnet matrix [23]. Protein variability server (PVS) was used to calculate protein variability index using Wu-kabat Variability coefficient [24]. From the multiple sequence alignment where the highest number of identical and similar amino acid and no gap was found, the sequence was selected as a conserved region. That conserved region was then used for antigenic site prediction.

### Detection of immunogenicity of conserved peptides

To evaluate the immunogenicity of the conserved peptides, various bioinformatics algorithms and computational tools were used. Bepipred (v1.0) [29] and B cell epitope prediction tools of The Immune Epitope Database (IEDB) [28] were used for this purpose. Bepipred predicts linear B-cell epitopes using hidden Markov model [29]. Default threshold 0.35 was used for Bepipred analysis. Among B cell epitope prediction tools of IEDB, prediction of linear epitopes from protein sequence tool was used. The Immune Epitope Database (IEDB) linear epitope prediction tools [28] made the option of using different prediction methods. Finally Kolaskar and Tongaonkar Antigenicity method [27] was applied in this study using a threshold of 1.000 because it predicts the antigenicity of the provided protein sequence. The epitopes which were found to be fully or at least 90% overlap between IEDB B-cell epitope prediction tool [28] and Bepipred prediction [29] are chosen as desired epitope sequences.

### Prediction of surface accessible epitopes

To predict the surface accessible epitope of the conserved peptide, Emini surface accessibility prediction tool [30] of the B cell epitope prediction tools of The Immune Epitope Database (IEDB) [28] was used for this purpose using default threshold level 1.0.

### Prediction of epitope conservancy

The epitope conservancy analysis tool from the IEDB analysis resource was employed for epitope conservancy prediction [31] of all predicted epitopes. The conservancy level of the epitopes were calculated by searching for identities in the given protein sequence.

### Prediction of epitope hydrophilicity

The conserved epitope was then also analyzed to determine the hydrophilicity of the predicted epitopes. Parker hydrophilicity prediction tool [32] of Immune Epitope Database (IEDB) [28] was used for this purpose and default threshold 3.448 was used.

### Prediction and evaluation protein 3D model

As the experimental structure of RNA directed RNA polymerase protein of any human coronavirus isolate was not found in protein data bank (PDB), a 3D structure was predicted using I-TASSER server [33]. I-TASSER server gives protein 3D structure by multiple threading alignments [33]. I-TASSER provided top models quality was then verified by PROCHECK software [34]. The model for which G factor was highest, and amino acid residues in favorable region was higher in PROCHECK analysis was selected as the best model. This model was then used to locate the epitope by using UCSF Chimera [35] visualization tool.

## Abbreviations

HCoV: Human coronavirus; SARS: Severe acute respiratory syndrome; SARS-CoV: SARS coronavirus; MERS-CoV: Middle east respiratory syndrome coronavirus; HCoV-229E: Human coronavirus 229E; HCoV-OC43: Human coronavirus OC43; HCoV-NL63: Human coronavirus NL63; HCoV-HKU1: Human coronavirus HKU1; ACE2: AngiotensinI-converting enzyme 2; DPP4: Dipeptyl peptidae 4.

## Competing interests

The authors declare that they have no competing interests.

## Authors’ contributions

RS & AI performed the analysis. AI conceived the project idea. RS and AI wrote the manuscript. Both authors read and approved the final manuscript.

## Supplementary Material

Additional file 1: Figure S1 Multiple sequence alignment of Spike (S) protein: Multiple sequence alignment of total 17 numbers of sequences of human coronaviruse isolates indicate that there is no conservation in their spike protein. This alignment was visualized by Jalview 2.8 [25] and color scheme used is Clustalx. Conservation showed here is based on 11 base scales where yellow color bar and star sign indicates the full conservation. Alignment quality was based on BLOSUM 62 substitution matrix score where yellow color indicates good quality. All the colors changes according to the conservation and alignment quality. Black bars showed the consensus sequence. Click here for file

Additional file 2: Figure S2 Multiple sequence alignment of envelope (E) protein: Figure legend as in supplementary Figure S1. Click here for file

Additional file 3: Figure S3 Multiple sequence alignment of membrane (M) protein: Figure legend as in supplementary Figure S1. Click here for file

Additional file 4: Figure S4 Multiple sequence alignment of nucleocapsid (N) protein: Figure legend as in supplementary Figure S1. Click here for file

Additional file 5: Figure S5 Conserved peptide found in RNA directed RNA polymerase by multiple sequence alignment of replicase polyprotein 1ab: All human coronaviruses are found to be conserved in their replicase polyprotein 1ab. Click here for file

Additional file 6: Figure S6. Ramachandran plot for RNA directed RNA polymerase protein: Red colored region is the most favored region, brown and yellow colored regions are additionally allowed region and generously allowed regions respectively. Click here for file

Additional file 7: Table S1. Sequence sources and other sequence related information. Click here for file
