# Supplementary figures and images for: A highly conserved WDYPKCDRA epitope in the RNA directed RNA polymerase of human coronaviruses can be used as epitope-based universal vaccine design
Source: BMC Bioinformatics. 2014 May 29;15:161. doi: 10.1186/1471-2105-15-161 (PMC4041900; doi:10.1186/1471-2105-15-161)

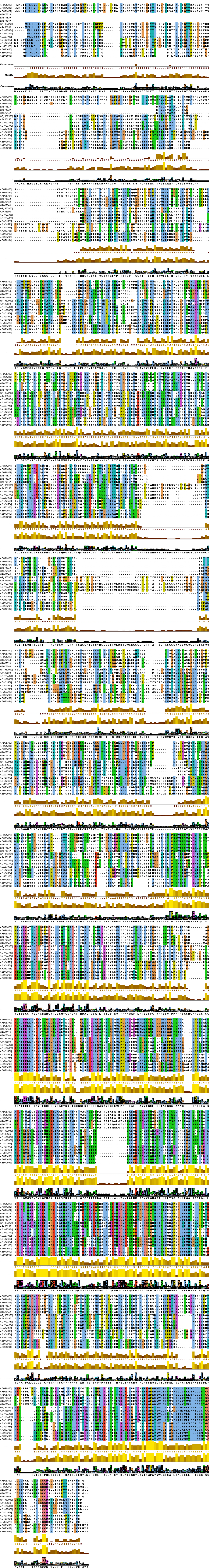

Supplement: Additional file 1: Figure S1 — Multiple sequence alignment of Spike (S) protein: Multiple sequence alignment of total 17 numbers of sequences of human coronaviruse isolates indicate that there is no conservation in their spike protein. This alignment was visualized by Jalview 2.8 [25] and color scheme used is Clustalx. Conservation showed here is based on 11 base scales where yellow color bar and star sign indicates the full conservation. Alignment quality was based on BLOSUM 62 substitution matrix score where yellow color indicates good quality. All the colors changes according to the conservation and alignment quality. Black bars showed the consensus sequence. [file 1471-2105-15-161-S1.jpeg]

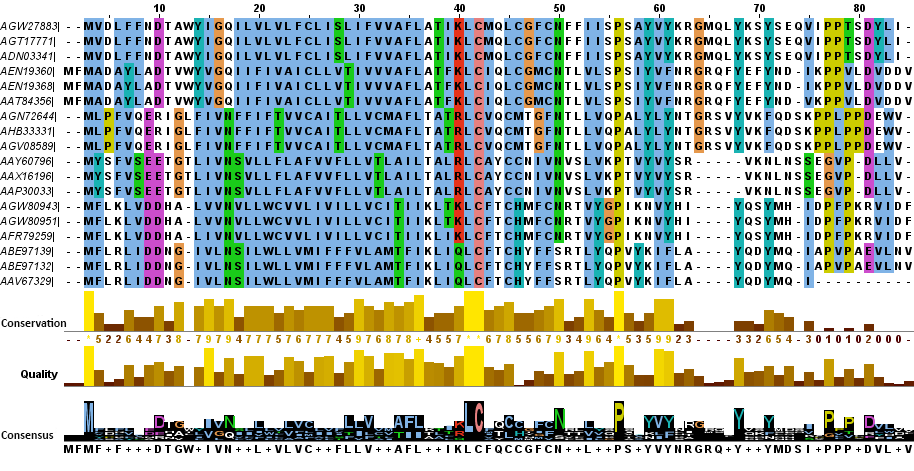

Supplement: Additional file 2: Figure S2 — Multiple sequence alignment of envelope (E) protein: Figure legend as in supplementary Figure S1. [file 1471-2105-15-161-S2.tiff]

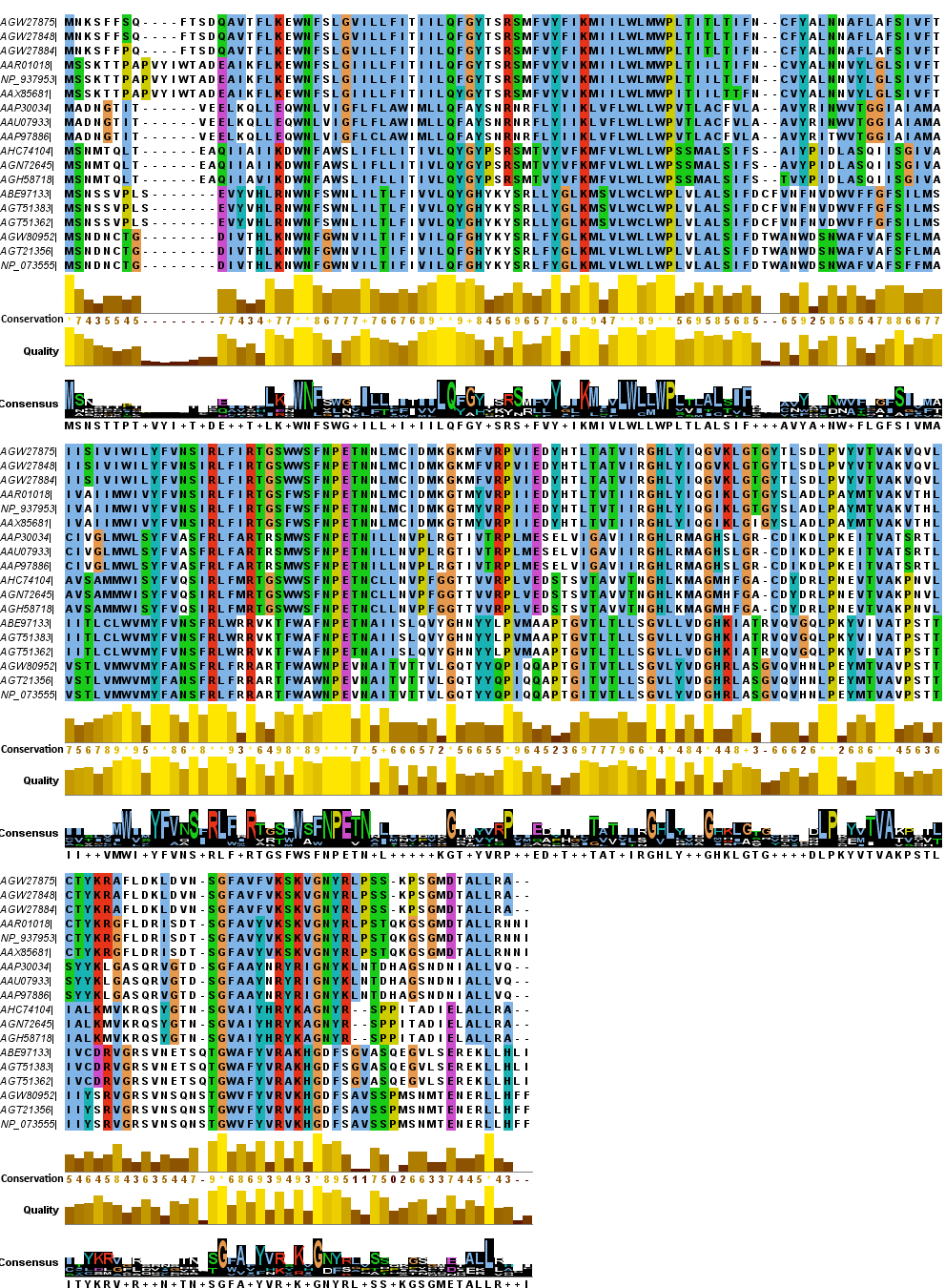

Supplement: Additional file 3: Figure S3 — Multiple sequence alignment of membrane (M) protein: Figure legend as in supplementary Figure S1. [file 1471-2105-15-161-S3.tiff]

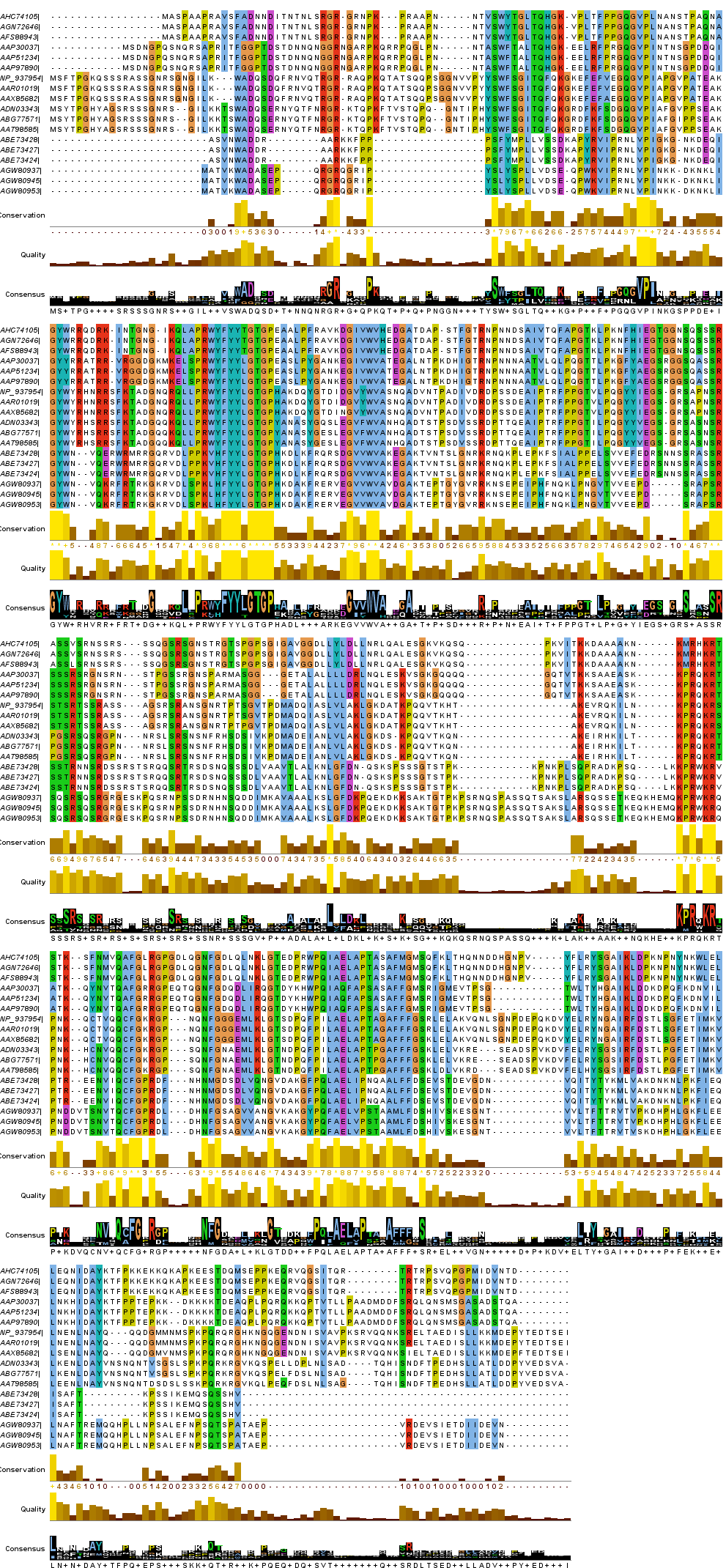

Supplement: Additional file 4: Figure S4 — Multiple sequence alignment of nucleocapsid (N) protein: Figure legend as in supplementary Figure S1. [file 1471-2105-15-161-S4.tiff]

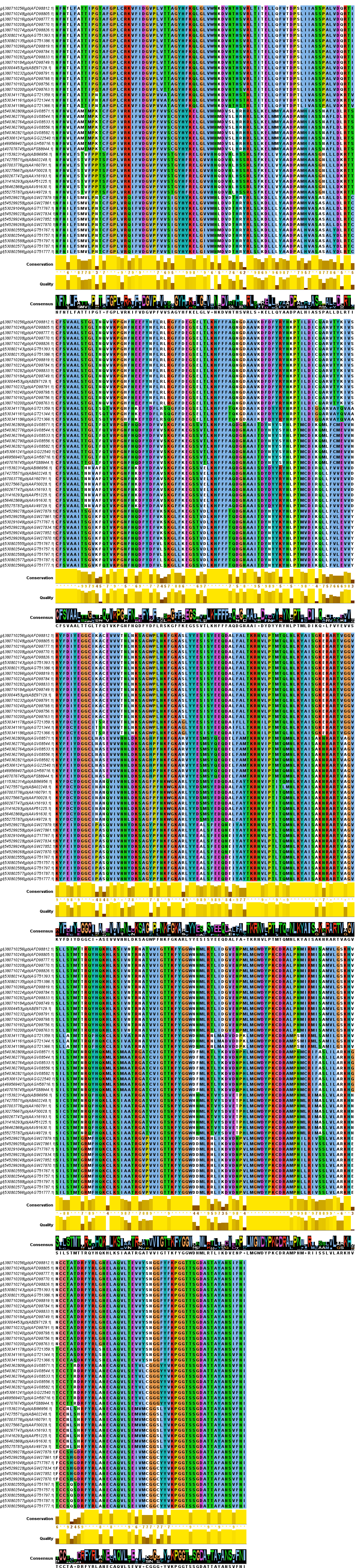

Supplement: Additional file 5: Figure S5 — Conserved peptide found in RNA directed RNA polymerase by multiple sequence alignment of replicase polyprotein 1ab: All human coronaviruses are found to be conserved in their replicase polyprotein 1ab. [file 1471-2105-15-161-S5.tiff]

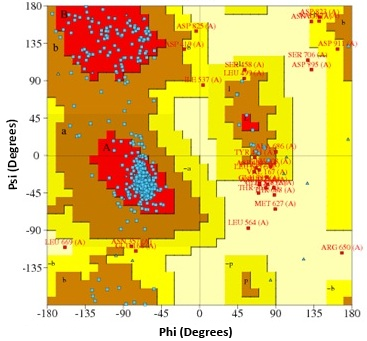

Supplement: Additional file 6: Figure S6. — Ramachandran plot for RNA directed RNA polymerase protein: Red colored region is the most favored region, brown and yellow colored regions are additionally allowed region and generously allowed regions respectively. [file 1471-2105-15-161-S6.tiff]
